# Artificial neural network-assisted prediction of radiobiological indices in head and neck cancer

**DOI:** 10.3389/frai.2024.1329737

**Published:** 2024-04-05

**Authors:** Saad Bin Saeed Ahmed, Shahzaib Naeem, Agha Muhammad Hammad Khan, Bilal Mazhar Qureshi, Amjad Hussain, Bulent Aydogan, Wazir Muhammad

**Affiliations:** ^1^Department of Physics, Florida Atlantic University, Boca Raton, FL, United States; ^2^Gamma Knife Radiosurgery Center, Dow University of Health Sciences, Karachi, Pakistan; ^3^Radiation Oncology, McGill University, Montreal, QC, Canada; ^4^Radiation Oncology, Aga Khan University Hospital, Karachi, Pakistan; ^5^Tawam Hospital, Al Ain, United Arab Emirates; ^6^Radiation and Cellular Oncology, University of Chicago, Chicago, IL, United States

**Keywords:** tumor control probability, normal tissue complication probability, head and neck cancer, artificial neural network, radiation therapy

## Abstract

**Background and purpose:**

We proposed an artificial neural network model to predict radiobiological parameters for the head and neck squamous cell carcinoma patients treated with radiation therapy. The model uses the tumor specification, demographics, and radiation dose distribution to predict the tumor control probability and the normal tissue complications probability. These indices are crucial for the assessment and clinical management of cancer patients during treatment planning.

**Methods:**

Two publicly available datasets of 31 and 215 head and neck squamous cell carcinoma patients treated with conformal radiation therapy were selected. The demographics, tumor specifications, and radiation therapy treatment parameters were extracted from the datasets used as inputs for the training of perceptron. Radiobiological indices are calculated by open-source software using dosevolume histograms from radiation therapy treatment plans. Those indices were used as output in the training of a single-layer neural network. The distribution of data used for training, validation, and testing purposes was 70, 15, and 15%, respectively.

**Results:**

The best performance of the neural network was noted at epoch number 32 with the mean squared error of 0.0465. The accuracy of the prediction of radiobiological indices by the artificial neural network in training, validation, and test phases were determined to be 0.89, 0.87, and 0.82, respectively. We also found that the percentage volume of parotid inside the planning target volume is the significant parameter for the prediction of normal tissue complications probability.

**Conclusion:**

We believe that the model has significant potential to predict radiobiological indices and help clinicians in treatment plan evaluation and treatment management of head and neck squamous cell carcinoma patients.

## 1 Introduction

Head and neck squamous cell carcinoma (HNSCC) is becoming a significant global health concern, with an estimated 30% increase in incidence rates by 2030 as per Global Cancer Observatory (GLOBOCAN) data, anticipating it to become a major global burden in time (Johnson et al., 2020). Radiation therapy (RT) is one of the modalities used for the management of HNSCC cases, often combined with surgery or chemotherapy making it an important modality in the treatment of HNSCC (Kawashita et al., 2020). The success of RT treatment relies on various factors, including the tumor’s stage, grade, treatment time, nutrition status, and treatment volume, as well as considerations such as tumor hypoxia (Huang and O’Sullivan, 2017). Additionally, the role of biological and radiological markers is evolving with clinical prognostic factors that play a crucial role in influencing treatment success (Konings et al., 2020; Peng et al., 2021).

In oncology, treatment outcomes revolve around two key factors: survival and toxicity. Survival assesses the effectiveness of interventions, measuring overall and progression-free durations (Abola et al., 2014). Simultaneously, managing and mitigating treatment-related toxicity is vital for the patient’s well-being and quality of life (Hunter et al., 2013).

The process of radiotherapy involves several crucial steps, with the planning stage serving as a pivotal checkpoint. During this phase, meticulous consideration is given to optimizing the delivery of radiation, ensuring maximum efficacy in targeting the tumor while minimizing potential harm to surrounding healthy tissues (Gardner et al., 2019). This planning step plays a foundational role in shaping the overall success and precision of radiotherapy treatment. The basic principle of planning is optimizing dose delivery to maximize the dose to the planning target volume (PTV) while minimizing harm to nearby tissues and organs at risk (OAR). Insufficient tumor coverage in radiotherapy can compromise treatment effectiveness, while overly reducing side effects may result in inadequate tumor treatment. Striking the right balance between optimal tumor control and minimizing side effects is crucial in the planning and implementation of radiotherapy. This delicate equilibrium is a key consideration for clinicians to ensure successful outcomes. This emphasizes the need for a nuanced approach to balancing the benefits and potential risks associated with radiotherapy (Pereira et al., 2014).

Like other treatment modalities, RT does come with known specific side effects (Dilalla et al., 2020). Despite leaps of advancements in radiation therapy and its standards, managing OAR toxicity remains a big frontier. Accurate risk estimation is crucial. For this purpose, integrating radiobiological indices like Tumor Control Probability (TCP) and Normal Tissue Complication Probability (NTCP) into treatment planning can quantify the response to irradiation, facilitating informed decision-making. The preference for one over the other creates a seesaw effect, leading to a trade-off where the loss of one aspect results in the gain of another (Yorke, 2023). Although precautionary measures are taken during both the planning and delivery stages, it is challenging to completely avoid toxicity to OAR. Dose-volume histograms (DVHs) conventionally describe 3D-dose distribution but have limitations. Integrating radiobiological indices with DVH, such as TCP and NTCP, provides a more comprehensive evaluation of treatment plans. Presently, the calculation methodologies for Tumor Control Probability (TCP) and Normal Tissue Complication Probability (NTCP) in radiation therapy heavily depend on radiobiological models. Specifically, the poison-based model estimates TCP for a given radiation dose, and the Lyman-Kutcher-Burman (LKB) model utilizes Dose-Volume Histogram (DVH) data to assess the likelihood of normal tissue complications (Warkentin et al., 2004). However, these models exhibit limitations as they oversimplify the intricate biological processes involved, overlooking factors such as tumor heterogeneity, organ- or tissue-specific mechanisms, and patient characteristics. To address these shortcomings, ongoing research direction is toward enhancing these models by integrating uncertainties, thereby promoting more nuanced clinical judgment in the context of treatment planning (Bufacchi et al., 2020).

In recent years, advancements in prediction outcomes have been notable, marked by the emergence of data-driven models (Isaksson et al., 2020). These models integrate dose-volume metrics, advanced bioinformatics tools, and disease- or patient-based prognostic factors. Several publications underscore the efficacy of these prediction models, demonstrating medium to good accuracy in forecasting clinical outcomes for head-and-neck cancer (Parmar et al., 2015; Giraud et al., 2019; Mahmood et al., 2021). These outcomes also encompass HNSCC high-grade RT toxicity including xerostomia, sticky saliva, radiation-induced trismus, and sensorineural hearing loss (Thor et al., 2021; Araújo et al., 2023). Concurrently, ongoing research aims to refine traditional radiobiological models by incorporating uncertainties for improved clinical judgment in treatment planning, addressing the complexity of biological processes and patient-specific factors (Gronberg et al., 2023).

The emergence of the machine learning in healthcare for making informed decisions, leading to effective patient management. The artificial neural network (ANN) showed significant progress in areas of outcome prediction, risk quantification personalized treatments, and diagnosis accuracy for both chronic and infectious diseases (Muhammad et al., 2019; Shafiq et al., 2022; Ataei et al., 2023). This also motivated us to utilize ANN in the current study.

This study aims to develop ANN for the prediction of radiobiological indices in HNSCC. The primary objective is to empower treatment planners with patient-specific information on TCP and NTCP, facilitating a more informed approach to prioritizing PTV and OAR during the treatment planning and evaluation stages. The proposed methodology involves utilizing an artificial neural network, a type of machine learning model, trained on a carefully curated dataset encompassing dose-volume metrics, patient-specific factors, and outcomes. ANN will be designed to predict radiobiological indices, particularly TCP and NTCP. Rigorous validation of the dataset will ensure the robustness of the model. The development of ANN for predicting radiobiological indices holds the potential to revolutionize HNSCC treatment planning. By providing treatment planners with patient-specific information, this approach aims to enhance the overall quality, efficacy, and outcome of radiation therapy, ultimately contributing to improved patient care and management.

## 2 Materials and methods

### 2.1 Data sets

Set I: The computed tomography (CT) images of 215 HNSCC patients were acquired from the publicly available repository “The Cancer Imaging Archive” (TCIA) (Clark et al., 2013). Patients were treated with curative intent RT from 2003 to 2013 at the University of Texas MD Anderson Cancer Center (Grossberg et al., 2018). The dataset comprises de-identified DICOM images, RT structures, planning parameters, DVH, and dose deposition data. The collection also consists of patient demographics, tumor grade, and stage, risk factors, recurrence, and survival information.

Set II: The dataset consists of CT scans of 31 HNSCC patients. Three CT scans were acquired for each patient throughout the treatment course. These include treatment planning, mid-treatment, or interim and post-treatment (i.e., follow-up) scans. Patients were treated with volumetric arc therapy (VMAT) at the Department of Radiation Oncology, the University of Miami Miller School of Medicine from 2011 to 2017 (Bejarano et al., 2019). Along with DICOM images, patient demographics, tumor histology, outcomes, RT structures, DVH, and planning parameters were available for public download at TCIA.

### 2.2 Methodology

**Figure 1:**
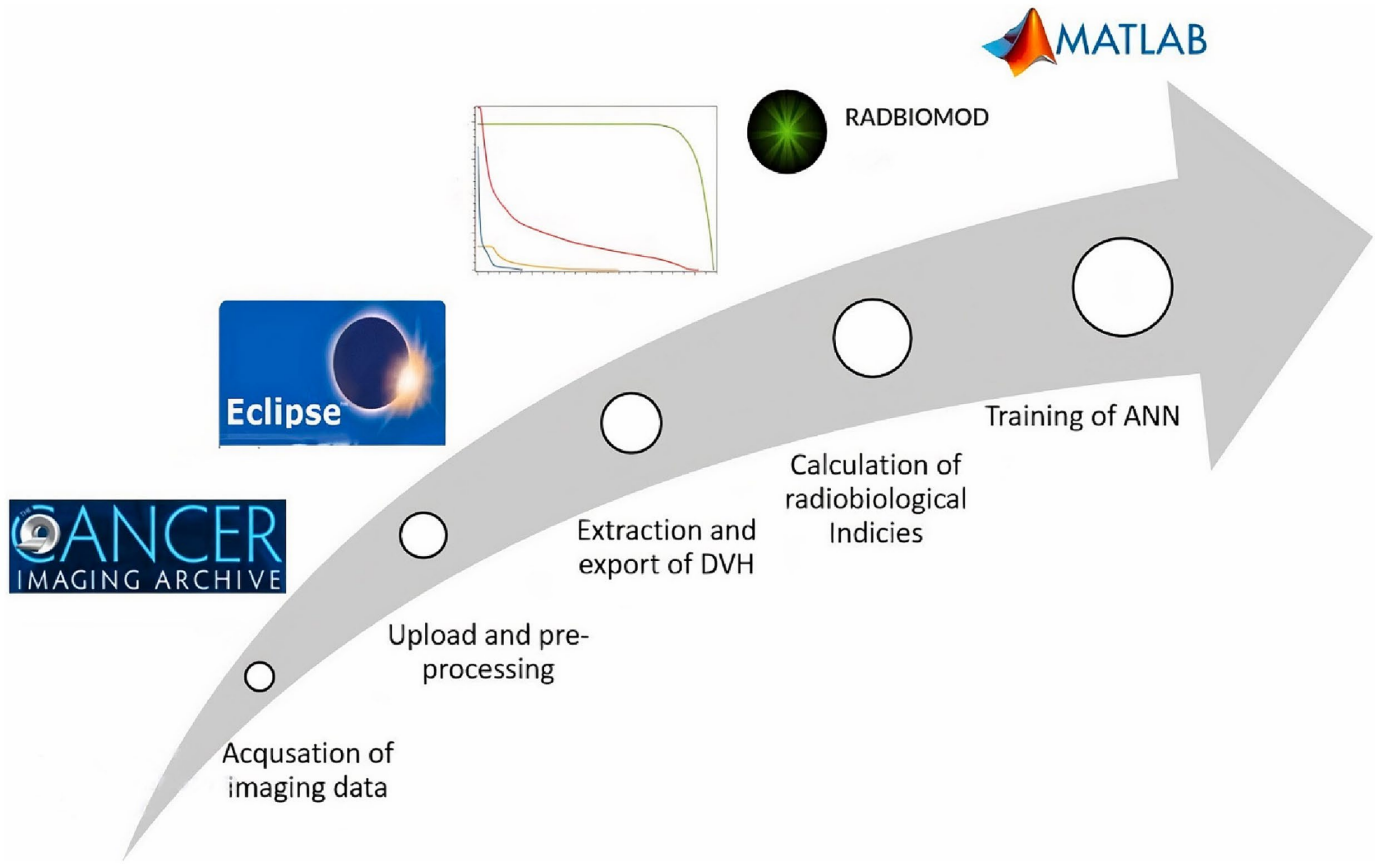
The methodology and basic components utilized for the development of ANN.

The process begins with the acquisition of imaging datasets from TCIA, followed by the upload to the treatment planning system for verification and pre-processing. Both parotid OARs and PTV were selected for DVH data extraction from the treatment planning system and then processed toward a spreadsheet for the calculation of radiobiological indices. Patient particulars along with radiobiological indices were used for the training of the neural network (Figure 1).

### 2.3 Data acquisition

TCIA is the repository for clinical and imaging data. It hosts various data collections of cancer, and non-cancer patients, and phantom studies. We selected two different sets of HNSCC patients which have RT structures, RT dose, and DVH information along with imaging and patient demographics. An open-source software “*NBIA Data Retriever V8.0*” was used to download both sets containing DICOM files and spreadsheets of clinical information for all the patients.

### 2.4 Pre-processing

**Figure 2:**
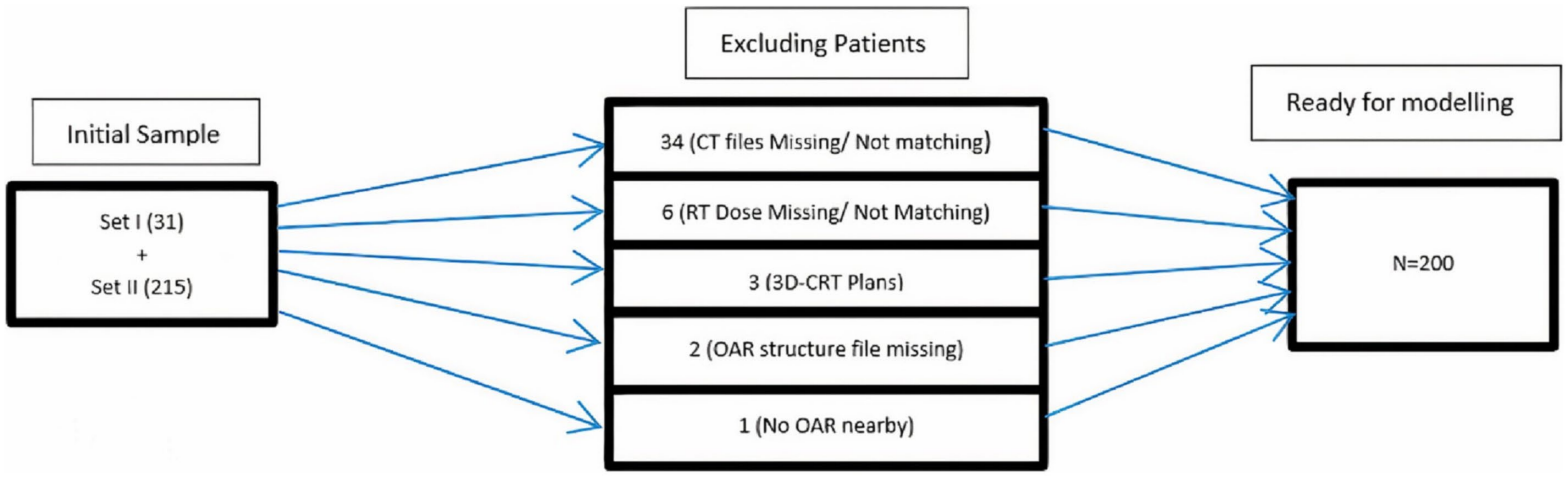
The inclusion/exclusion flow chart of patient datasets.

In Eclipse™ (treatment planning system) V 15.1, the anonymized DICOM files of both sets were uploaded with new IDs, i.e., HN001, and HN002. The CT images of the planning scan, RT structures, and RT dose files were imported to generate a dummy plan for each patient. These plans were evaluated for dose distribution in PTV and OARs. Verification and constancy of downloaded patient data were also ensured with these plans. Patients excluded from further processing were shown in a flow chart (Figure 2). Derived OAR structures were also contoured to extract dose parameters. These parameters along with others were used as input parameters for ANN. The last step in the pre-processing stage is the export of DVH of PTV, OAR, and derived structures in tabular format for the calculation of indices.

### 2.5 Radiobiological indices

RADBIOMOD® (V. beta 0.3) is a spreadsheet-based software tool for the calculation of radiobiological indices and evaluation of RT treatment plans (Chang et al., 2016). It offers multiple radiobiological models for the calculation of TCP and NTCP. We used the Gay & Niemierko (Gay and Niemierko, 2007) model for the calculation of both TCP and NTCP of PTV and parotids, respectively. Gay & Niemierko’s model utilizes the equivalent uniform dose (EUD) mechanistic formulation based on the linear–quadratic cell survival model (Webb and Nahum, 1993). To supplement ANN, we also used the Poisson model (Jackson et al., 1995) and (Zaider and Minerbo, 2000) for the calculation of TCP and the Lyman–Kutcher–Burman model (Lyman, 1985) for NTCPs. The calculation begins with the import of Tabular DVH from each dummy plan in Eclipse to the RADBIOMOD. Dose fractionation, total dose, treatment duration, and α/β ratios, i.e., The proportional contribution of single and double-strand breaks to cell death, were also used as input for the calculation of TCP and NTCP. Then computed radiobiological indices for each patient were routed toward training of an artificial neural network.

### 2.6 Artificial neural network architecture

**Figure 3:**
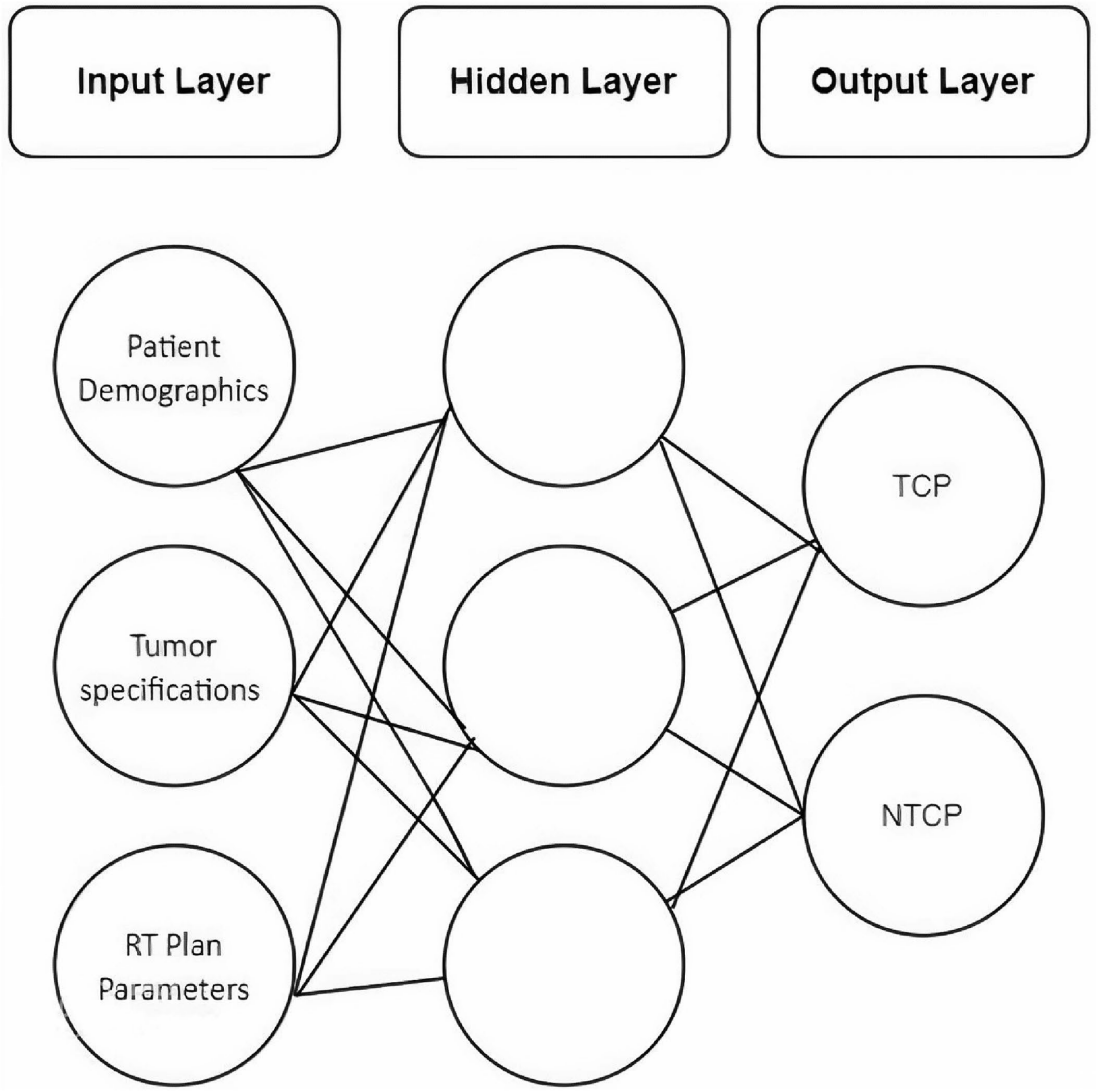
The architecture of feed-forward ANN.

A feed-forward neural network with back-propagation training was designed using a machine learning toolbox in Matlab® V.2022a. Under the umbrella of Neural Network Fitting, the network consists of three layers, i.e., input, hidden, and output layer (Figure 3).

Initially, there were 10 neurons in a hidden layer which were modified as needed during the training stage. Scaled Conjugate Gradient (SCG) was selected as a training function. SCG is a fully automated and fast supervised learning algorithm (Møller, 1993). The SCG algorithm updates the weights *W* of the neural network (Eq. 1).

(1) $$W_{k+1}=W_{k}-\alpha_{k}\nabla f\left(W_{k}\right)$$

Where $W_{k+1}$ is the weight matrix of the neural network at iteration *k* and $\alpha_{k}$ is step size, which can be adaptively adjusted during the optimization process. The gradient $f\left(W_{k}\right)$ is typically computed using backpropagation, which efficiently calculates the gradient of the error function with respect to each weight in the network. The distribution of training, validation, and testing cohorts are 75, 15, and 15%, respectively. For progress monitoring and validation checks, the maximum number of training epochs used as stopping criteria as per mean squared error (MSE) is defined as follows (Eq. 2).

(2) $$MSE=\frac{1}{N}\overset{i=N}{\underset{i=1}{\sum }}{\left(y_{i}-x_{i}\right)}^{2}$$

Where $x_{i}$ is the input value, $y_{i}$ represents the predicted value and N is the total number of datasets. Error histograms and regression plots were used to determine the error distribution and accuracy of ANN at different stages.

#### 2.6.1 Predictors

The input cohort used for the machine learning process was a combination of all eligible patients from Set-I and Set-II, i.e., *N* = 200. There were 28 patient-specific input variables majorly comprised of demographics, tumor stage, and site, RT-dose, and treatment planning parameters. Subclasses of predictors include the type of concurrent chemotherapy drug administered with RT, radiation dose indices to target and OAR, percentage volumes of parotids lying inside the target, and patients’ weight loss after RT.

#### 2.6.2 Outcomes

The output layer consists of TCP values calculated via Poisson, Zaider-Minerbo, and EUD methods. NTCP calculated for both parotids by Lyman-Kutcher-Burman and EUD methods were also included in the output layer.

## 3 Results

**Figure 4:**
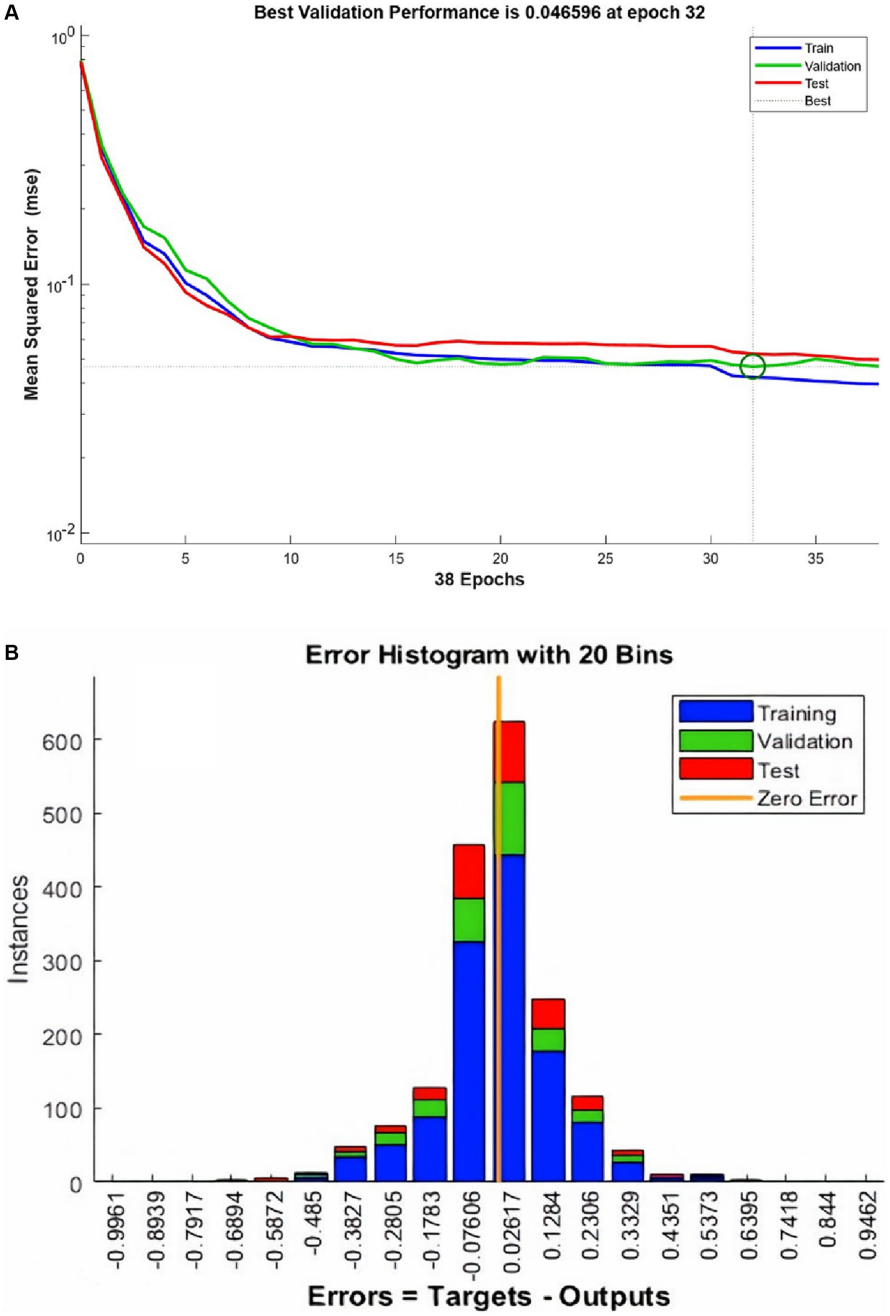
The performance plot **(A)** for ANN and error histogram **(B)** between predicted and target values.

ANN training begins without the hardware acceleration of the personal computer and it performs efficiently. The training and validation performance evaluated by MSE vs. no. of the epochs plot (Figure 4A). The plot shows a gradual decrease in MSE values versus the number of epochs. The progressive decline in MSE values represents the good accuracy of training and test data. Further, it shows the adjustment of the right weight after every epoch. The MSE reduces around 15 times during the training duration and after reaching the saturation the training stops at epoch number 38. It took 1.06 s to complete the training. The best validation performance was noted at epoch number 32 with an MSE value of 0.0465.

**Figure 5:**
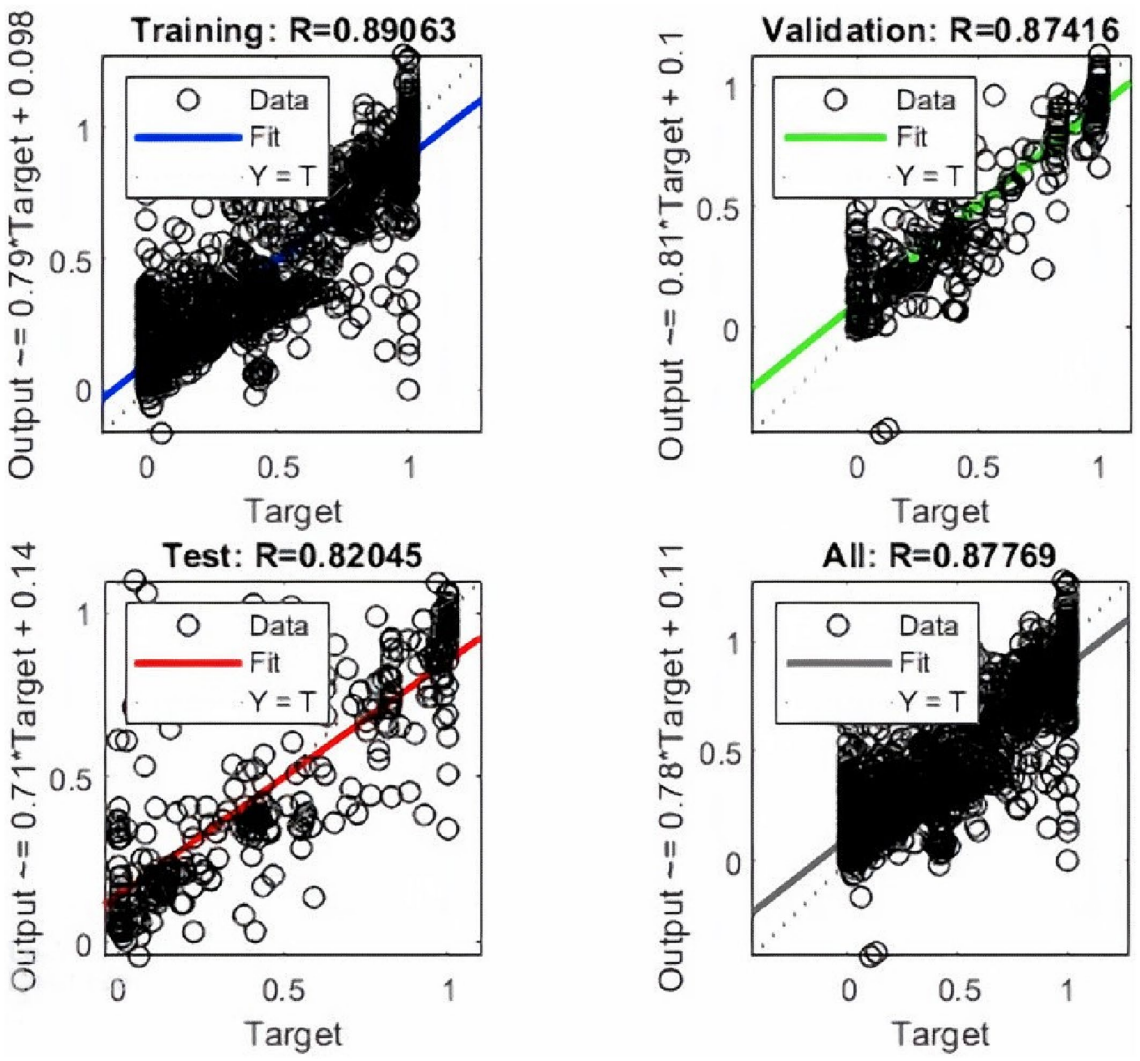
The linear regression plots for the training, validation, and testing stages of the ANN.

An error histogram was plotted to determine the distribution of errors between the predicted and target values at different instances (Figure 4B). The histogram was also used as a measure of the prediction performance of ANN. Error-values were distributed in 20 bins and placed in columns. The error range for the model lies between −0.9961 and 0.9462. More than 75% of the data points from training, validation, and testing lie in the error range of −0.076 to 0.1258. After the validation checks, the prediction capacity of the model was accessed with 15% of the test cases (Figure 5). Linear regression plots between target and output showed for training, validation, and testing stages, represented by blue, green, and red lines, respectively. Each plot is supported by a line fit equation between the predicted and target values where the target is the independent variable and the output is a dependent variable. The proportionality between target and output is the coefficient of a target for which the value on the stage of training is 0.79 and 0.81, 0.71 for the validation and testing stages, respectively. The constants at the end of equations were errors or residuals and its incorporation scaled the target to make better predictions in output. The error values were 0.098, 0.1, and 0.14 for the training, validation, and testing stages. These equations highlight the performance of ANN. The regression coefficient (R) represents the accuracy of the model. At the final stage, the value of R found for all stages together was 0.877 and 0.89, 0.87, 0.82, respectively, for the training, validation, and testing stages.

## 4 Discussion

For the prediction of radiobiological indices, datasets were chosen with few special considerations for the development of ANN. (1) The patients were treated with similar conformal RT techniques and narrow integral and per fraction dose variations. (2) Both or ipsilateral parotid must be nearby or involved with the PTV. (3) DVH must be available for both parotids and PTV. The above principles help maintain consistency among input variables and result in smooth progression during the training stage.

The particular significance given to parotid glands as an OAR in this study of HNSCC patients is because even partial sparing of these glands will have a positive effect on patients’ quality of life (QOL) (Deasy et al., 2010). Our ANN model showed the capacity to predict NTCPs for parotid glands and TCPs for target structure. This tool will equip clinicians to make informed decisions with patient-specific information to adapt and modify OAR and PTV structures, which may result in better QOL for patients.

The inclusion of patient data from different institutions strengthens the model and incorporates the inter-variability of treatment planning and contouring practices among the institutes. It also enables the model to adapt further data sets from different regions and origins to enhance the learning and prediction process. Additionally, the architecture of ANN was designed in a way that makes it susceptible to adding more OARs from HNSCC patients.

The data sets used for training and validation were of conventional fractionation, ranging from 2 to 2.12 Gy. This limits the prediction of indices for hypofractionation plans. The non-availability of comprehensive data on the radiobiological effects of higher doses on OARs was one of the limiting factors. However, a substantial portion of HNSCC patients in developing and developed countries are still treated with conventional fractions, which highlights the significance of this prediction model.

Radiobiological indices were calculated from different models to complement the network with the latest available organ-specific radiobiological parameters for HNSCC RT. It enables the prediction model for current and upcoming HNSCC datasets to be tested and comprehended. In contrast to open source available calculators like RADBIOMOD and RBMODELV1 (Patel et al., 2022) which utilize tabular DVH values for the calculation of TCP and NTCP, the proposed ANN model predicts the radiobiological indices by incorporating significant parameters like tumor specifications, RT parameters, DVH and patient demographics. This makes the current model a more comprehensive predictor of TCP and NTCP.

The ANN-driven prediction model with its primary application to assist treatment planners and radiation oncologists with TCP and NTCP values, would potentially minimize the see-saw effect between coverage of PTV and sparing of parotids during RT treatment planning of HNSCC. This could empower radiation oncologists to optimize the margins of PTV near parotids in conjunction with their TCP and NTCP values. Another edge it could provide in adaptive RT by incorporating previous and current treatment plan parameters into the model and producing TCP and NTCP for insight.

For future work, the incorporation of more HNSCC OARs such as the spinal cord, cochlea, and optical structures from current datasets is in process. We are also seeking larger datasets with variable dose fascinations to scale up the performance of the current model.

## 5 Conclusion

In conclusion, our study has successfully developed ANN with a high prediction accuracy (*R* = 0.82) for radiobiological indices, offering a valuable tool for HNSCC treatment planning. The observed correlation between parotid volume within the PTV and increased NTCP underscores the importance of minimizing PTV margins to reduce post-irradiation complications. Furthermore, our research expanded predictive factors to include demographics, tumor characteristics, treatment parameters, and additional variables such as concurrent chemotherapy, radiation dose indices, and post-RT weight loss. These enhancements, combined with DVH, TCP, and NTCP models, contribute to the development of personalized and more accurate tools for toxicity prediction, advancing the goal of optimizing cancer care for HNSCC patients.

## Data availability statement

The original contributions presented in the study are included in the article/supplementary material, further inquiries can be directed to the corresponding author.

## Author contributions

SA: Data curation, Formal analysis, Investigation, Methodology, Validation, Writing – original draft, Writing – review & editing. SN: Data curation, Methodology, Writing – review & editing. AK: Data curation, Investigation, Methodology, Writing – review & editing. BQ: Data curation, Investigation, Methodology, Writing – review & editing. AH: Data curation, Formal analysis, Investigation, Methodology, Writing – review & editing. BA: Data curation, Formal analysis, Methodology, Writing – review & editing. WM: Conceptualization, Data curation, Funding acquisition, Methodology, Project administration, Resources, Supervision, Validation, Visualization, Writing – review & editing.
